# “What If Others Think I Look Like…” The Moderating Role of Social Physique Anxiety and Sex in the Relationship between Physical Activity and Life Satisfaction in Swiss Adolescents

**DOI:** 10.3390/ijerph20054441

**Published:** 2023-03-02

**Authors:** Silvia Meyer, Christin Lang, Sebastian Ludyga, Alexander Grob, Markus Gerber

**Affiliations:** 1 Department of Psychology, University of Basel, Missionsstrasse 62, 4055 Basel, Switzerland; 2 Department of Sport, Exercise, and Health, University of Basel, Grosse Allee 6, 4052 Basel, Switzerland

**Keywords:** life satisfaction, physical activity, social physique anxiety, sex, adolescents

## Abstract

Background: Physical activity has been shown to have a positive effect on life satisfaction in adolescents. Despite these benefits, physical activity levels constantly drop during adolescence, suggesting potential interfering factors in this link. Since worries about physical appearance are an important issue at this age, this study aims to examine the relationship between physical activity and life satisfaction in adolescents and explores possible moderating effects of social physique anxiety and sex. Methods: We used data from a longitudinal study with *N* = 864 vocational students (mean age = 17.87 years, range: 16–25, 43% female) from Switzerland. To test our hypotheses, we used multiple hierarchical regression analyses as well as simple slope analyses. Results: We did not find a significant direct effect of physical activity on life satisfaction. However, we found a significant two-way interaction between physical activity and social physique anxiety. An additional significant three-way interaction occurred, indicating that a positive effect of physical activity on life satisfaction holds only for female adolescents with low social physique anxiety levels. Conclusions: This study highlights the importance of developing a healthy relationship with one’s body to fully benefit from physical activity, especially for female adolescents. Taken together, these results reveal important considerations for physical activity educators.

## 1. Introduction

Life satisfaction plays a crucial role during adolescence in reaching developmental milestones and in ensuring a healthy transition into adulthood [1]. Among adolescents, life satisfaction represents a key indicator for psychological health and psychological wellbeing [2]. Therefore, it is not surprising that well-being, in general, is associated with a variety of positive personal, behavioral, psychological, and social factors at this age [3].

Life satisfaction is defined by a global evaluation of one’s life and represents the cognitive–evaluative component of subjective well-being (SWB) [4]. It is well-known that life satisfaction levels decrease during adolescence, particularly between the ages of 11 and 16 years [5,6,7]. This decrease can be explained by the various challenges that adolescents face during puberty and the tempo at which these changes come along [8]. Further, decreases in domain-specific life satisfaction (family, hobbies/leisure time, social life) have been observed across adolescence, which may contribute to a decrease in global life satisfaction [5,6].

Life satisfaction is an important factor for psychological functioning and plays a crucial role in the risk of development of emotional and behavioral problems, such as depression or anxiety [9,10]. Accordingly, low levels of life satisfaction increase other behavioral health risk factors, such as substance abuse, which potentially increase the risk of developmental problems [2]. It is therefore crucial to consider potential explaining factors to better understand the decrease in life satisfaction during this age.

### 1.1. The Role of Physical Activity

Physical activity has gained a lot of attention as a protective factor during adolescence [11]. Physical activity is not only beneficial for adolescents’ physical health [12] but also has a positive impact on psychological factors such as self-esteem, emotions, or mood [13,14]. A link between engagement in regular physical activity and better psychosocial health and well-being in adolescents has repeatedly been confirmed (for reviews see: [15,16,17]). As part of psychosocial health, life satisfaction is linked to physical activity as well as to different forms of physically active behaviors, such as exercising or participating in a sports club [18]. Several reviews have shown that higher levels of life satisfaction (or happiness) are associated with higher levels in various physical activity outcomes, such as higher exercise levels or more active lifestyles [17,19]. Additionally, a recent study with a large representative sample of adolescents in 44 countries reported a significant association between physical activity and life satisfaction [20].

The relevance of this relationship becomes even more clear by looking at the impact of a lack of physical activity. Thus, physical inactivity during adolescence can track into adulthood [21] and increase the risk of obesity [22]. Moreover, adolescents who do not engage in vigorous physical activity show significantly lower levels of life satisfaction [23]. To avoid these possible negative consequences associated with a lack of physical activity, it seems crucial to focus on and obtain a better understanding of the mechanisms and possible amplifying factors of regular physical activity.

Most researchers agree on the fact that it is probably a combination of both psychological and neurobiological factors that lead to benefits of physical activity on psychosocial health and well-being [24,25]. In their recent review, Rose and Soundy [15] proposed that physical activity enhances autonomy development. Autonomy then, on the one hand, has a direct effect on adolescent’s well-being and, on the other hand, increases intrinsic motivation to spend more time in physical activity. In an earlier review, Paluska and Schwenk [25] also discussed theory-based mechanisms; in line with the self-efficiency theory, they suggested that exercise can lead to feelings of self-confidence and a sense of success, which directly improves mood and also leads to more confidence to use available resources important for mental health. Finally, another review concluded that the strongest evidence was found for the physical self-perception mechanism; thereby, physical activity leads to improvements in physical self-perception, which enhances self-esteem and thus well-being of adolescents [11,26].

### 1.2. The Role of Social Physique Anxiety

Despite the potential beneficial effects of physical activity on adolescents’ life satisfaction and well-being, physical activity levels tend to decline during adolescence [27]. Accordingly, several interpersonal as well as structural factors exist that drive adolescents to quit or avoid physical activity [28]. For instance, research has shown that girls reported losing interest, insufficient time, and a perceived lack of competence as the main reasons for quitting sports [29].

As highlighted in the previous chapter, improved self-perceptions are one of the strongest explanatory factors for the relationship between physical activity and life satisfaction [11]. In line with this, social physique anxiety could be an important contributing factor to explain why some adolescents do not sufficiently engage in physical activity [30]. Social physique anxiety is defined as the anxiety of being evaluated negatively by others based on one’s physique and physical appearance (e.g., body fat, muscularity, tone, body proportions) and therefore can be seen as a connection between a person’s image of his/her body and the satisfaction or dissatisfaction with this image [31]. This notion was supported by empirical evidence showing that significant associations exist between body dissatisfaction and social physique anxiety [32] and that especially weight- and muscular-related social comparisons are predictive of the development of social physique anxiety in college students [33].

During adolescence, the body undergoes a series of significant changes [34], including changes in body proportions or body fat distribution [35]. At the same time, physical attractiveness becomes more important for adolescents [36]. Due to the high importance of physical appearance and the changes that come with puberty, there is an increased risk of developing a negative body image during adolescence [37]. This could also be due to the fact that, during this developmental stage, social comparison is an important tool for adolescents to develop their self-concept [38]. Combined with the insecurities that come along with puberty-related body changes, adolescents are usually more sensitive about the way their bodies are evaluated by others [39,40]. Girls seem to be at particularly high risk of social comparison as well as negative self-perceptions of their bodies. They show a significant difference between their perceived and desired body perceptions, independent of their BMI [41].

Negative body perceptions in adolescents are not only frequent but also correlated with high social physique anxiety levels [42]. Due to adolescents’ strong focus on social comparisons and more specifically on how their body is seen or evaluated by others, social physique anxiety has been related to participation motives, engagement, and avoidance of physical activity in adolescents [43]. Due to their lower body image, teens might be anxious about others thinking negatively about their bodies or may have low perceptions of their physical competence and therefore may stop engaging in physical activities such as exercise and sport [28,39]. Further, many adolescents with social physique anxiety may have had negative experiences related to past physical activities (which may be perpetuated by their anxiety); as the literature shows, it is how physical activity is perceived that motivates or discourages an individual from engaging in further physical activity [44].

There are several further theories that explain why people may stop engaging in regular physical activity [45]. One of these theories is the theory of impression management and self-presentation [46]. The theory assumes that if people are motivated to make certain impressions but doubt they will be successful (such as the fear of being negatively judged in terms of their body), they usually experience anxiety and behave rather shyly. More importantly, they use strategies to minimize the likelihood of being evaluated in an unflattering way. Therefore, not engaging in physical activity can be understood as a coping strategy to avoid potential negative impressions [46].

Studies have shown that adolescents use both behavioral and cognitive avoidance of physical activity to cope with social physique anxiety, with girls also using more emotion-focused coping strategies (e.g., behavioral avoidance or appearance management) compared to boys [39,47]. Apart from leading to reduced physical activity levels, social physique anxiety is also directly linked to mental health and life satisfaction levels [48]. A study with male recreational cyclists and triathletes (aged 18–60 years) showed that social physique anxiety negatively impacted their life satisfaction through a decrease in perceived satisfaction with basic psychological needs [49]. In summary, since social physique anxiety leads to avoidance of physical activity and is also directly linked to lower life satisfaction, it could be reasoned that adolescents with social physique anxiety might have had negative experiences while participating in physical activity [44] and might therefore not benefit from it in the same way as adolescents who have healthy relationships with their bodies.

### 1.3. The Role of Sex

Evidence suggests that girls and boys show significant differences in how they see and evaluate their physical self-concepts [50]. For instance, girls seem to be more concerned about being overweight and show a higher desire for thinness, while boys often aim to be more muscular, even though this might lead to gaining weight [51]. Male adolescents usually have more positive self-perceptions regarding their bodies as well [42,52]. In line with this, female adolescents score higher on social physique anxiety than their male counterparts across the entire lifespan (for a review see: [43]).

Moreover, there is ample evidence that girls have lower levels of physical activity in general and also show a stronger decrease in physical activity from childhood to adulthood [53], whereas boys show higher and more constant levels of physical activity during this transition [42]. Moreover, boys also show higher levels in general and health-related life satisfaction than girls [5,54].

Besides these differences in physical activity, life satisfaction, and social physique anxiety itself, there are indicators that there are sex differences in the associations. Thus, the negative relationships between physical activity and mental health as well as physical activity and life satisfaction have been found to be stronger in girls [55,56]. However, it remains largely unknown whether other relationships between these constructs, especially potential moderating effects, also differ between boys and girls.

### 1.4. Purpose of This Study

As summarized above, past research has shown different relationships between physical activity and life satisfaction in adolescents and connected social physique anxiety to both physical activity and life satisfaction separately. Sex differences were also investigated before but focus mainly on the differences in the investigated constructs itself. Consequently, little is known of how these different effects are all connected. The present study aims to better understand the mechanisms involved in the relationship between physical activity and life satisfaction in adolescence and therefore investigates if this relationship is moderated by social physique and sex.

Given the literature presented above, we hypothesized that physical activity is related to higher life satisfaction, both at present and 10 months later (Hypothesis 1) (e.g., [19]). We also expected this effect to be only significant if social physique anxiety levels are low (Hypothesis 2) [43,49]. Finally, we assumed that, for girls, the moderating effect of social physique anxiety is stronger than for boys (Hypothesis 3) [43,53,56].

## 2. Materials and Methods

### 2.1. Participants

We used data from the EPHECT study (Effects of a Physical Education-based Coping Training), a cluster randomized trial. Vocational students from two schools in Switzerland were recruited and asked to complete a battery of questionnaires during a physical education lesson at the beginning (t1: September/October 2010) and at the end of the academic year (t2: May/June 2011). During the data assessment, two research assistants were present. Students were assured confidentiality and provided informed written consent. In addition, minors (students below 18 years of age) provided written parental informed consent. The study was approved by the local ethics committee and was conducted in line with the guidelines set forth in the Declaration of Helsinki. Data from this study have been published previously, mainly focusing on the effect of stress in combination with physical activity and mental health outcome [57,58,59].

A total of *N* = 1242 students participated in the baseline (t1) data assessment. The dropout rate from baseline (t1) to follow-up (t2) was 30.4% (*N* = 378). However, we did not find any statistically significant differences in physical activity, life satisfaction, social physique anxiety, or the distribution of sex between participants who dropped out and peers who completed both data assessments. The final sample with complete longitudinal data was composed of *N* = 864 participants (comprising 43% female; which is representative of the sex distribution among students in vocational training in Switzerland (41% female students in 2020, see: [60])).

### 2.2. Measures

#### 2.2.1. Physical Activity

A short form of the International Physical Activity Questionnaire (IPAQ-SF) [61] was used to assess physical activity. Questions included the number of days in the last week the students were vigorously or moderately physically active for at least 10 minutes. Vigorous physical activity was defined as movements that cause one to work up a sweat, such as jogging, biking, or playing soccer. Moderate physical activity was defined as movements causing one to be just a little short of breath, such as dancing, table tennis, or cycling at an easy pace. We summed these two items up to an overall moderate-to-vigorous physical variable, representing the days on which participants were physically active. The IPAQ has shown good validity for adolescents above 15 years of age and has shown to predict physical activity levels comparably to the accelerometer [62,63,64].

#### 2.2.2. Life Satisfaction

Three items (those with the highest factor loadings in the initial studies, see [65,66]) of the Satisfaction With Life Scale (SWLS) [67] were used to measure life satisfaction. Students were asked to evaluate the following questions: (a) “In most ways, my life is close to my ideal.” (b) “I’m satisfied with my life.” (c) “So far I have gotten the important things I want in life.” Answers were given on a 7-point Likert scale ranging from 1 (*completely incorrect*) to 7 (*completely correct*). The three items showed good internal consistency in the present sample (Cronbach’s α = 0.77) and were combined to build a mean score. The validity of the SWLS score has been demonstrated repeatedly in previous studies [67,68]. Specifically, the three-item scale of the SWLS has shown similar validity to the original five-item scale [69].

#### 2.2.3. Social Physique Anxiety

Social physique anxiety was measured with the Social Physique Anxiety Scale (SPAS) [31]. We used the 9-item version of the scale, which has shown acceptable validity [70,71], where students had to evaluate questions such as “I worry that there are parts of my body that people may not like.” Answers were given on a 5-point Likert scale ranging from 1 (*not at all*) to 5 (*extremely*). Two items were reverse coded and had to be recoded before calculating the mean score. In the present sample, the index showed good internal consistency (Cronbach’s α = 0.82).

### 2.3. Statistical Analysis

Descriptive and bivariate correlation analyses with all the main study variables were calculated in the first step. Body mass index (BMI) was measured as weight in kilograms divided by height in meters squared. Students were asked to self-report their height (without shoes) in cm and weight (without clothes) in kilograms. With these answers, the BMI was calculated. We included age, body mass index (BMI), and group (intervention vs. control condition) as covariates to ensure the results would be independent of participants’ age, BMI, or whether they participated in the intervention or not. We then also controlled for baseline life satisfaction levels in the prospective analyses. Further analyses were controlled for socio-economic status, assessed via self-reported financial situation of the adolescents (see Table S1 in the Supplemental Materials). Since these covariate analyses did not change overall effects, we decided to report these data as supplemental online material only.

To test the moderating effect of social physique anxiety and sex on the relationship between physical activity and life satisfaction, we used conditional process modeling to examine three-way interactions. In the first step, we analyzed the data at the first measurement point in a cross-sectional way to detect the proposed associations. For these analyses, the full sample (students with complete baseline data) was used. In the second step, we then analyzed the longitudinal data by adding the data from the second measurement point to investigate a potential direction and the robustness of the effects. For these analyses, only students with complete data at baseline and follow-up were considered. We calculated a moderated moderation (Model 3) using the PROCESS macro developed by Hayes [72] in SPSS. PROCESS estimates the best fitting ordinary least squares regression model and examines the interactions. We first performed cross-sectional and then longitudinal analyses. Therefore, we defined life satisfaction as the outcome variable and added physical activity as the predictor variable. Social physique anxiety and sex were then added as moderator variables. The Johnson–Neyman technique was used to derive regions of significance for the two-way interaction (PA × SPA) at a value of the third variable (sex).

We mean-centered the independent variable (PA) and the linear moderator variable (SPA) before entering them into the regression model. The results of the regression models are presented as unstandardized regression coefficients (*b*), standard errors, and *p* values. To further interpret the slopes of the three-way interaction effects and plot the interactions, we used simple slope analyses and the plotting procedures [73]. To this end, the moderator variable “sex” was divided into binary categories: boys (1) and girls (2). The level of significance was set at *p* < 0.05 across all analyses.

## 3. Results

### 3.1. Descriptive Statistics and Bivariate Correlations

Table 1 provides an overview of the sample characteristics and descriptive statistics of the main study variables as well as the main covariates. Statistics are presented separately for the full sample (with complete baseline data) and the prospective sample (participants with complete data at baseline and follow-up). Means, standard deviations, minimal and maximal values, as well as skewness and kurtosis are presented.

The bivariate correlations between the investigated variables are presented in Table 2. Again, correlations are presented separately for the full sample with valid cross-sectional data at baseline (above diagonal) and those participants with both complete baseline and follow-up data (below diagonal). Physical activity was positively correlated with life satisfaction, whereas a negative correlation occurred between physical activity and social physique anxiety. Social physique anxiety was negatively correlated with life satisfaction. Boys reported significantly higher levels of physical activity and lower levels of social physique anxiety than girls. Age was significantly and negatively correlated to physical activity and positively correlated with BMI. Finally, BMI was positively correlated with social physique anxiety.

### 3.2. Two- and Three-Way Interactions

To examine interaction effects, we calculated a cross-sectional process model (Table 3). We first calculated the analyses with the full sample which included complete baseline data (Sample 1: *N* = 1242), and then with those participants who had full baseline and follow-up data (Sample 2: *N* = 864). In the cross-sectional analyses, after adjustment for age, BMI, and group, a significant main effect appeared for social physique anxiety (Sample 1: *b* = −0.466, *p* < 0.001, Sample 2: *b* = −0.372, *p* < 0.001) on life satisfaction in both samples. In contrast, no significant main effect was found for physical activity (Sample 1: *b* = 0.029, *p* = 0.094, Sample 2: *b* = 0.027, *p* = 0.197). In the larger sample, a significant two-way interaction was found between physical activity and social physique anxiety (*b* = −0.045, *p* = 0.029). In the smaller sample, this two-way interaction effect did not reach statistical significance, although the sign of the interaction pointed in the same direction (*b* = −0.023, *p* = 0.298). No significant three-way interaction between physical activity, social physique anxiety, and sex was found in either sample. The variables included in the cross-sectional model explained a total of 11% (Sample 1) and 10% (Sample 2) of variance in life satisfaction.

To further explore the potential three-way interaction, we calculated a prospective conditional process model. As shown in Table 4, students with higher baseline life satisfaction and participants assigned to the control group reported higher life satisfaction at follow up. No statistically significant main effects were found for age, sex, and BMI.

**Figure 1:**
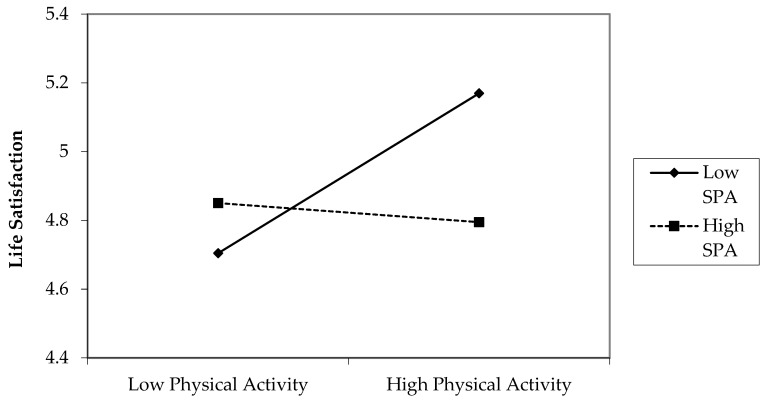
Two-way interaction of physical activity (baseline) and social physique anxiety (baseline) on life satisfaction (follow-up) for both sexes. Notes: SPA = social physique anxiety.

After controlling for baseline life satisfaction and covariates, there were no significant main effects for physical activity or for social physique anxiety. However, there was a statistically significant (negative) two-way interaction between physical activity and social physique anxiety (*b* = −0.052, *p* = 0.013), indicating that only adolescents with low social physique anxiety benefitted from higher physical activity in relation to their life satisfaction (see Figure 1). In the longitudinal analyses, a significant three-way interaction between physical activity, social physique anxiety, and sex appeared (*b* = 0.080, *p* = 0.014). The Johnson–Neyman technique showed that the relationship between physical activity and life satisfaction was only significant for girls who had low social physique anxiety (1 SD below the mean). The variables included in the prospective model explained a total of 27% of variance in life satisfaction at follow-up.

### 3.3. Simple Slope Analyses

**Figure 2:**
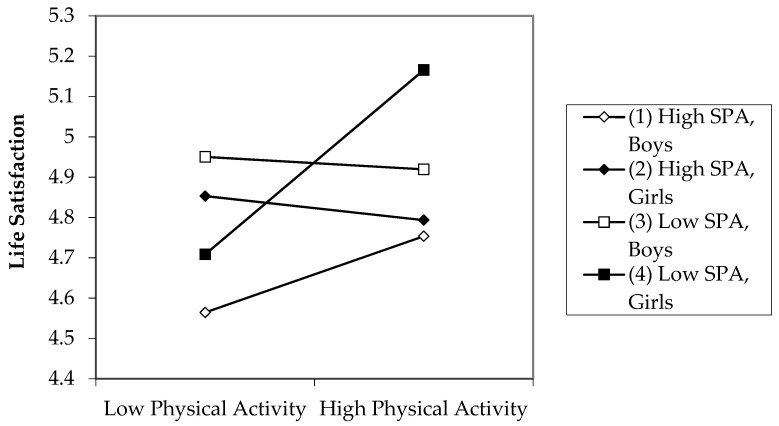
Three-way interaction of physical activity (baseline), social physique anxiety (baseline), and sex on life satisfaction (follow-up). Notes: SPA = social physique anxiety.

Simple slope analyses were used to interpret and to compare the slopes of the three-way interaction of physical activity, social physique anxiety, and sex when predicting adolescents’ life satisfaction at follow-up. As displayed in Figure 2, different pattern occurred for boys and girls. Girls with low social physique anxiety reported higher life satisfaction when they were more physically active (*b* = 0.071, *p* = 0.003). For girls with high social physique anxiety, no significant effect of physical activity on life satisfaction occurred (*b* = −0.005, *p* = 0.984). For boys, the results point in a different direction, but the slopes for boys were not statistically significant, indicating that physical activity had no significant effect on life satisfaction among boys, independent of their social physique anxiety levels.

Slope difference tests further pointed toward significant differences between the girls with high and the girls with low social physique anxiety slope (*t*(864) = −2.662, *p* = 0.008). These results indicate that if social physique anxiety levels are high, girls seem to not fully benefit from physical activity.

## 4. Discussion

The goal of this study was to investigate the role of social physique anxiety and sex on the relationship between physical activity and life satisfaction in adolescents. In the first step, we analyzed the data cross-sectionally with a larger sample. In the second step, we analyzed the data longitudinally to get a better understanding of the potential direction of the effects.

Contrary to our first hypothesis, we did not observe a significant effect of physical activity on life satisfaction, neither cross-sectionally nor longitudinally. One reason for the statistically non-significant finding could be that physical activity impacts rather emotional components of well-being, such as positive affect and happiness, than the cognitive components reflected by life satisfaction. This is in line with a recent large international study with young adolescents (aged 10 to 12 years), which showed a stronger correlation between physical activity and positive affect than with cognitive measures of subjective well-being, including life satisfaction [74]. Another assumption is that the link between physical activity and life satisfaction only becomes relevant in adulthood. This notion is in line with that of Maher et al. [75], who reported that physical activity and life satisfaction were correlated in middle and late adulthood, but not in young adulthood, which the authors explained could be due to an increased perception of control. However, the fact that the effects were not significant can as well be explained by the existence of other moderating factors. Therefore, we postulated with our second hypothesis that social physique anxiety moderates the effect between physical activity and life satisfaction.

Our data support our hypothesis, as shown with a significant two-way interaction between physical activity and social physique anxiety. The effect was found in the cross-sectional analyses and was corroborated in the longitudinal analyses. Simple slope analyses revealed that the effect of physical activity on life satisfaction was only significant when social physique anxiety levels were low. In other words, engagement in physical activity had no positive effect on life satisfaction among adolescents who were challenged with social physique anxiety. This result is in line with a study of 298 young adolescents (mean age: 11.6 years) showing that social physique anxiety was associated with lower enjoyment in sports as well as reduced leisure-time physical activity [76]. A study with 245 female college students (mean age: 19.9 years) further found that enjoyment of sports is crucial to benefit from psychological effects, for example, gaining a higher self-esteem through sporting activities [77]. Moreover, another study with 1492 adolescents showed that enjoyment of sports is even more important than the frequency of the physical activities to increase self-esteem [78]. Yet, self-esteem is subsequently linked to higher life satisfaction levels in adolescents [54]. In contrast, concerns about physical appearance or the judgment of others on their bodies have been shown to reduce participation in sports among adolescents [79,80]. At the same time, adolescents with low levels of physical activity and sedentary behavior report lower levels of life satisfaction [16].

Another assumption for the moderating effect of social physique anxiety on the link between physical activity and life satisfaction is that for adolescents with high social physique anxiety, situations surrounding physical activity, for example, changing clothes or a shared shower, may constitute a source of distress [81]. These and other forms of stress then lead to a decrease in adolescents’ life satisfaction [30,82].

Finally, the question arises whether this moderating effect presents differently depending on the sex of the adolescents. In line with our third hypothesis, a significant association between physical activity and life satisfaction was only present if participants were female and social physique anxiety levels low. Several reasons exist to explain this three-way interaction. First, girls tend to be more concerned about people staring or even laughing at their appearance during sports or of being called names that refer to their weight [79]. Therefore, girls may experience more distress while participating in physical activities, as they perceive higher levels of teasing from peers and show higher withdrawal rates in sports clubs than boys [79,83]. Second, boys and girls may use different coping strategies when experiencing social physique anxiety. Eklund and Bianco [81] argued that social physique anxiety can have controversial effects on physical activity, as it can lead to avoidance because of the fear of being evaluated or increased levels through the motivation of changing their physical appearance (for example, to become more muscular or to lose body fat). It is also possible that boys who have high levels of social physique anxiety use physical activity to come closer to the image they want others to have of their bodies. This has also been confirmed in previous research showing that a higher percentage of girls use behavioral avoidance to cope with social physique anxiety compared to boys. In turn, the percentage of boys using physical activity as a coping strategy was higher [47]. In addition, among boys, contrary to girls, a significant correlation between physical activity and perceived body attractiveness was found, indicating that physical activity may improve their physical self-perception [84]. Our results point in a similar direction, as life satisfaction was slightly higher among boys with social physique anxiety if they were highly engaged in physical activities (see Figure 2). However, simple slope effects were not significant for boys, so the findings have to be interpreted with caution.

This study has several strengths such as the use of longitudinal data, the large sample size, and instruments that all showed satisfactory internal consistency. Moreover, we used two- and three-way interactions as well as simple slope analyses to determine the relationship between physical activity, life satisfaction, social physique anxiety, and sex, which allowed us to examine moderating effects. Despite these strengths, some shortcomings should not remain unmentioned. Limitations include the exclusive focus on self-reported data during a physical education lesson, which could have added stress to students who already have social physique anxiety or could have led to social desirability or recall bias; the fact that we only focused on frequency when assessing physical activity; and the focus on adolescents in vocational education and training, which makes it difficult to generalize the findings to a broader student population. It is also noteworthy that in the longitudinal analyses, we found a main effect for group showing that students assigned to the control group reported higher life satisfaction at follow-up. The reasons for this unexpected result are not entirely clear, particularly as the intervention had no impact on stress and coping, the key outcome variables of the intervention study [59].

Further research should focus on representative samples of adolescents to gain more information about the generalizability of these effects. More than two measurement points would allow one to figure out how the measured variables change over time and how these changes predict each other. From a methodological point of view, it would be interesting to include other physical activity measures beyond self-reported data such as pedometers or actigraphs. Furthermore, this study highlights the importance of getting a better understanding of adolescents’ relationships with their bodies when examining the health-enhancing potential of physical activity. Future replication studies should examine whether different constructs such as body image, weight perception, or body satisfaction moderate or have differential effects on the association between physical activity and life satisfaction. For intervention studies, it seems important to consider potential social physique anxiety levels beforehand and test intervention effects in this topic for girls as well. Lastly, the increasing presence of social media could also be an important factor to consider while helping students dealing with body dissatisfaction. Social media use has shown to be associated with body dissatisfaction in adolescents; however, it was also found that positive parent relationships can prevent adolescents from those effects [85]. On the other hand, approaches such as the body positivity movement on social media, where mainly pictures that might be different from societal beauty ideals were posted, have also been shown to help adolescents to develop a healthy relationship with their own bodies [86]. Further studies therefore could include the use of social media as an additional moderating factor.

## 5. Conclusions

The present study gives new insights into the moderating role of social physique anxiety on the relationship between physical activity and life satisfaction in adolescents. Specifically, our data suggest that physical activity-based interventions are beneficial for promoting adolescent’s physical and mental health, yet only if they do not struggle with high levels of social physique anxiety. Thus, it seems crucial for adolescents to develop a more positive relationship with their body, particularly girls with social physique anxiety.

Moreover, our findings highlight the fact that adolescent girls and boys cope differently with body-related anxieties or worries. Coaches and teachers who interact with adolescents during physical activity should take these differences into consideration. For instance, by addressing and discussing the concerns and coping strategies students may have regarding body dissatisfaction as well as the potential effects of social media.

## Figures and Tables

**Table 1:** Descriptive statistics of the main study variables.

	Sample 1: Complete Data at Baseline (*N* = 1242)						Sample 2: Complete Data at Baseline and Follow-Up *(N* = 864)					
Variables	*M*	*SD*	Min	Max	Skew	Kurt	*M*	*SD*	Min	Max	Skew	Kurt
Age	17.98	1.36	16	25	1.057	2.600	17.87	1.32	16	25	1.113	2.764
BMI	22.24	3.22	13.85	50.76	2.005	10.764	22.14	2.97	16.38	41.03	1.302	3.351
Physical activity (baseline)	5.13	3.22	0	14	0.509	−0.141	5.11	3.25	0	14	0.522	−0.074
Social physique anxiety (baseline)	2.38	0.72	1	5	0.769	0.446	2.39	0.72	1	5	0.755	0.393
Life satisfaction (baseline)	4.87	1.18	1	7	−0.675	0.298	4.89	1.14	1	7	−0.628	0.186
Life satisfaction (follow-up)	4.87	1.16	1	7	−0.673	0.355	4.87	1.16	1	7	−0.673	0.355

**Table 2:** Bivariate correlations (Pearson, two-tailed) between study variables.

Variables	1.	2.	3.	4.	5.	6.
1. Physical activity (baseline)		−0.15 ***	0.31 ***	0.01	0.13 ***	−0.09 **
2. Social physique anxiety (baseline)	−0.15 ***		−0.25 ***	0.22 ***	−0.31 ***	0.01
3. Sex (boys = 1, girls = 0)	0.31 ***	−0.29 ***		0.17 ***	0.08 **	0.01
4. BMI (baseline)	−0.02	0.27 ***	0.16 ***		−0.06 *	0.18 ***
5. Life satisfaction (baseline)	0.17 ***	−0.28 ***	0.09 **	−0.09 **		−0.05
6. Age (baseline)	−0.14 ***	0.02	0.01	0.18 ***	−0.05	
7. Life satisfaction (follow-up)	0.11 **	−0.19 ***	0.04	−0.07 *	0.51 ***	−0.00

Notes: Values above diagonal represent correlations between variables in the full sample with complete baseline (t1) data (*N* = 1242). Values below the diagonal represent correlations between variables in the sample of participants with both complete baseline (t1) and follow-up (t2) data (*N* = 864). * p < 0.05. ** *p* < 0.01. *** *p* < 0.001.

**Table 3:** Cross-sectional conditional process model with life satisfaction at baseline as dependent variable, physical activity as predictor, and social physique anxiety and sex as moderators.

	Sample 1: Complete Data at Baseline (*N* = 1242)				Sample 2: Complete Data at Baseline and Follow-Up *(N* = 864)			
Variable	*b*	SE	*t*	*p*	*b*	SE	*t*	*p*
Intercept	5.322	0.447	11.894	<0.001	5.321	0.544	9.768	<0.001
Physical activity	0.029	0.017	1.674	0.094	0.027	0.021	1.291	0.197
Social physique anxiety	−0.466	0.069	−6.727	<0.001	−0.372	0.081	−4.613	<0.001
Sex (boys = 1, girls = 0)	−0.064	0.072	−0.895	0.371	−0.039	0.085	−0.462	0.645
Age	−0.030	0.024	−1.270	0.204	−0.022	0.029	−0.765	0.444
BMI	0.005	0.011	0.441	0.659	−0.002	0.014	−0.161	0.872
PA × SPA	−0.045	0.020	−2.179	0.029	−0.024	0.023	−1.042	0.298
PA × Sex	0.012	0.023	0.523	0.601	0.045	0.027	1.657	0.098
SPA × Sex	−0.123	0.095	−1.299	0.194	−0.151	0.113	−1.336	0.182
PA × SPA × Sex	0.051	0.030	1.685	0.092	0.064	0.036	1.797	0.073
*R* ^2^	0.113				0.104			
*F*	17.435 ***				10.993 ***			

Notes: PA = physical activity, SPA = social physique anxiety. *** *p* < 0.001.

**Table 4:** Longitudinal conditional process model with life satisfaction at follow-up as dependent variable, baseline physical activity as predictor, and baseline social physique anxiety and sex as moderators.

	Sample 2: Complete Data at Baseline and Follow-Up *(N* = 864)			
Variable	*b*	*SE*	*t*	*P*
Intercept	2.252	0.514	4.380	<0.001
PA	0.026	0.019	1.404	0.161
Social physique anxiety	−0.081	0.073	−1.109	0.268
Sex (boys = 1, girls = 0)	−0.090	0.076	−1.175	0.240
Life satisfaction	0.471	0.031	15.329	<0.001
Age	0.036	0.026	1.387	0.166
BMI	−0.008	0.012	−0.683	0.495
Group (intervention = 1, control = 0)	−0.230	0.0672	3.420	0.007
PA × SPA	−0.052	0.021	−2.504	0.013
PA × Sex	−0.011	0.024	−0.461	0.645
SPA × Sex	−0.129	0.101	−1.277	0.202
PA × SPA × Sex	0.079	0.032	2.464	0.014
*R* ^2^	0.279			
*F*	29.8976 ***			

Notes: PA = physical activity, SPA = social physique anxiety. *** *p* < 001.

## Data Availability

The data presented in this study are available on request from the corresponding author. The data are not publicly available due to privacy reasons.

## Supplementary Materials

The following supporting information can be downloaded at: www.mdpi.com/article/10.3390/ijerph20054441/s1, Table S1: Longitudinal conditional process model with life satisfaction at follow-up as dependent variable, baseline physical activity as predictor, and baseline social physique anxiety and sex as moderators.
