# Supplementary material for: “What If Others Think I Look Like…” The Moderating Role of Social Physique Anxiety and Sex in the Relationship between Physical Activity and Life Satisfaction in Swiss Adolescents
Source: Int J Environ Res Public Health. 2023 Mar 2;20(5):4441. doi: 10.3390/ijerph20054441 (PMC10002265; doi:10.3390/ijerph20054441)
Supplement: Supplementary file 1 [file ijerph-20-04441-s001.zip › ijerph-2229592-Supplemental Material.pdf]

Supplemental Material

Table S1. Longitudinal conditional process model with life satisfaction at follow-up as dependent variable, baseline physical activity as predictor, and baseline social physique anxiety and sex as moderators.

	Sample 2: Complete data at baseline and follow-up (*N* = 864)			
**Variable**	***b***	***SE***	***t***	***P***
Intercept	2.226	0.535	4.161	<.001
PA	0.026	0.019	1.391	.161
Social physique anxiety	-0.081	0.073	-1.108	.268
Sex (boys = 1, girls = 0)	-0.090	0.077	-1.175	.240
Life satisfaction	0.470	0.031	15.113	<.001
Age	0.036	0.026	1.393	.164
BMI	-0.008	0.012	-0.679	.498
Group (intervention = 1, control = 0)	-0.231	.067	-3.423	<.001
Familys fiancial situation	.008	.0454	0.182	.856
PA × SPA	-0.053	0.021	-2.506	.012
PA × Sex	-0.011	0.024	-0.448	.654
SPA × Sex	-0.128	0.101	-1.271	.204
PA × SPA × Sex	0.079	0.032	2.467	.014
R^2	.279			
F	27.378***			
